# Optimizing perioperative systemic dexamethasone use in total knee arthroplasty: a narrative review on current evidence

**DOI:** 10.1186/s43019-026-00336-2

**Published:** 2026-08-03

**Authors:** Jisu Park, Tae Woo Kim, Moon Jong Chang, Seung-Baik Kang

**Affiliations:** 1 Department of Orthopedic Surgery, SMG-SNU Boramae Medical Center, Seoul, Republic of Korea; 2 Department of Orthopedic Surgery, Seoul National University College of Medicine, Seoul, Republic of Korea; 3 Knee Center, Gangnam St. Peter’s General Hospital, Seoul, Republic of Korea

**Keywords:** Total knee arthroplasty, Corticosteroid, Dexamethasone, Perioperative pain management, Diabetes mellitus

## Abstract

**Background:**

Perioperative corticosteroids, particularly dexamethasone, have become a widely adopted component of multimodal pain management in total knee arthroplasty (TKA). While clinical practice guidelines strongly recommend their use, detailed guidance on optimal dosing, frequency, and safety in special populations, especially patients with diabetes mellitus, is still limited.

**Main text:**

This narrative review synthesizes the current evidence on systemic perioperative dexamethasone use in TKA, focusing on clinical questions: agent and route selection, dose optimization, dosing frequency, and safety, including considerations for patients with diabetes. A literature search of PubMed, EMBASE, and the Cochrane Library was conducted for studies published between January 2015 and April 2026. Randomized controlled trials, systematic reviews, meta-analyses, clinical practice guidelines, and observational studies addressing systemic corticosteroid use in primary TKA were included. Recent evidence supports intravenous (IV) dexamethasone as the most commonly studied and clinically practical systemic agent. A 16 mg dose provides greater early analgesic and antiemetic benefit than lower doses, and repeat dosing through postoperative day (POD) 1 is supported, with extension to POD 2 emerging but not yet definitive. Perioperative dexamethasone has not been associated with increased infection or wound complications. In patients with well-controlled diabetes, it appears safe with appropriate glycemic monitoring, although safety data are largely limited to doses of 8–10 mg.

**Conclusions:**

Current evidence supports IV dexamethasone as an effective component of multimodal analgesia after TKA. In the general TKA population, 16 mg appears more effective than lower doses for early postoperative pain, opioid consumption, and postoperative nausea and vomiting (PONV)-related outcomes. Repeat dosing through POD 1 is supported by available evidence, whereas extension to POD 2 remains promising but requires further validation. In patients with well-controlled diabetes, perioperative dexamethasone appears safe with appropriate glycemic monitoring, although safety data are largely limited to doses of 8–10 mg, and the safety of the 16 mg dose in this population remains insufficiently defined.

**Supplementary information:**

The online version contains supplementary material available at 10.1186/s43019-026-00336-2.

## Background

Total knee arthroplasty (TKA) is one of the most commonly performed orthopedic procedures worldwide, yet postoperative pain remains a significant barrier to early recovery and patient satisfaction. Multimodal analgesia has become the standard approach within enhanced recovery after surgery (ERAS) protocols, incorporating various pharmacologic agents with different mechanisms of action to optimize pain control while minimizing opioid-related complications [1].

Corticosteroids were initially introduced in the perioperative setting primarily for the prevention of postoperative nausea and vomiting (PONV), with doses as low as 4–5 mg of dexamethasone demonstrating sufficient antiemetic efficacy [2]. Subsequently, evidence emerged that higher doses could provide meaningful analgesic benefits, prompting a broader role for perioperative corticosteroids in pain management after TKA. The utilization of intraoperative dexamethasone in elective total joint arthroplasty (TJA) in the USA has increased from 46.3% in 2015 to 64.7% in 2020, reflecting this expanding clinical adoption [3].

In 2022, joint clinical practice guidelines from the American Association of Hip and Knee Surgeons (AAHKS), American Academy of Orthopedic Surgeons (AAOS), Hip Society, Knee Society, and American Society of Regional Anesthesia and Pain Medicine (ASRA) provided a strong recommendation for perioperative corticosteroid use in TJA, and the procedure-specific postoperative pain management (PROSPECT) working group similarly endorsed preoperative intravenous (IV) dexamethasone as part of the multimodal analgesic regimen for TKA [4, 5]. However, neither guideline specifies an optimal dose, number of doses, or duration of administration, nor provides differentiated recommendations for patients with diabetes mellitus (DM).

Since the publication of these guidelines, substantial new evidence has accumulated, including dose-comparison randomized controlled trials (RCTs), meta-analyses on dosing frequency, and prospective data on safety in patients with diabetes. This narrative review differs from previous reviews by focusing specifically on practical perioperative decision-making after TKA, including dose selection, repeat-dose strategy, and safety considerations in patients with diabetes.

## Main text

This narrative review was conducted in accordance with the scale for the assessment of narrative review articles (SANRA) recommendations [6].

### Literature search strategy

A structured literature search was conducted in PubMed/MEDLINE, EMBASE, and Cochrane Central Register of Controlled Trials (CENTRAL) for articles published between January 2015 and April 2026. The search strategy combined terms related to total knee arthroplasty, corticosteroid agents, and clinical outcomes, including pain, infection, and diabetes. The search was limited to English-language publications involving human subjects. Reference lists of included studies and relevant clinical practice guidelines were manually screened for additional articles. The detailed search strategy is presented in Additional file 1.

### Inclusion and exclusion criteria

Studies were included if they (1) were randomized controlled trials, systematic reviews with or without meta-analysis, clinical practice guidelines, or observational studies; (2) evaluated systemic perioperative corticosteroid administration in primary TKA; and (3) reported outcomes related to dose optimization, dosing frequency, analgesic efficacy, or safety, including in patients with DM. Studies exclusively evaluating periarticular corticosteroid injection without a systemic comparison arm were excluded. Case reports, conference abstracts, letters to the editor, and trial registry records were also excluded.

### Data extraction and synthesis

Data from the included studies were extracted narratively and organized according to main clinical questions: agent and route selection, dose optimization, dosing frequency, and safety, with particular attention to patients with DM. Key study characteristics, including study design, sample size, intervention, comparator, and primary findings, are summarized in Tables 1, 2, 3, 4, and 5. As this is a narrative review rather than a systematic review, a comprehensive risk of bias assessment and a quantitative meta-analytic synthesis were not performed; the level of evidence for each cited study is indicated in Table 1 to facilitate clinical interpretation. To support the recommendations, however, the methodological quality of the pivotal primary studies underlying these recommendations was appraised: the randomized trials were assessed using the Cochrane risk of bias 2 (RoB2) tool, and the comparative nonrandomized studies using the methodological index for nonrandomized studies (MINORS). These assessments are presented in Additional files 2 and 3. A total of 36 articles were included in the final review.

**Table 1:** Summary of studies on agent and route of administration

Study	Study type	LOE	Procedure	*N*	Comparison	Key findings
Hatayama et al. (2021) [9]	Single-center RCT	I	TKA	100	Periarticular triamcinolone versus IV dexamethasone	Periarticular triamcinolone gave lower pain scores than IV dexamethasone, both at rest (3.1 ± 2.0 versus 4.3 ± 2.1, *P* = 0.007) and during walking (4.9 ± 2.0 versus 6.3 ± 2.3, *P* = 0.002) at 24 h
Chan et al. (2023) [10]	Single-center RCT	I	TKA	178	Placebo versus IV dexamethasone alone versus periarticular triamcinolone alone versus combined IV + periarticular	IV and combined corticosteroid lowered pain compared with periarticular alone, both at rest (1.3 ± 1.2/1.3 ± 1.5/1.8 ± 1.7) and during knee flexion (3.8 ± 1.7/3.9 ± 2.0/4.4 ± 1.8); the IV-containing groups, but not periarticular alone, were better than placebo (rest *P* = 0.026; flexion *P* = 0.007). The combined regimen further improved rehabilitation versus placebo (greater muscle power, 4 versus 3, *P* = 0.001; longer walking distance, 43.0 m versus 24.8 m, *P* < 0.001)
Kwak et al. (2025) [11]	SR & Network MA	I	TKA	14 RCTs	Periarticular versus IV corticosteroid	Periarticular and IV corticosteroids did not differ in pain (activity pain SMD 0.09 [−0.23 to 0.42]) or opioid use (SMD 0.01 [−0.15 to 0.17]); periarticular gave better early resting pain (POD 1), whereas IV was superior for PONV (periarticular versus IV OR 3.16 [1.70–5.86])

*IV* intravenous, *LOE* level of evidence, *MA* meta-analysis, *OR* odds ratio, *POD* postoperative day, *PONV* postoperative nausea and vomiting, *RCT* randomized controlled trial, *SMD* standardized mean difference, *SR* systematic review, *TKA* total knee arthroplasty

**Table 2:** Summary of studies on dose optimization

Study	Study type	LOE	Procedure	*N*	Comparison	Key findings
Lex et al. (2021) [12]	SR & MA	I	TKA + THA	17 RCTs	IV dexamethasone > 15 mg versus < 15 mg	Activity-pain reduction was significantly greater with > 15 mg at both POD 0 (MD −3.26 versus −0.98, subgroup *P* = 0.006) and POD 1 (MD −1.66 versus −0.49, subgroup *P* = 0.023). PONV at POD 2 was also reduced more with > 15 mg (log OR −1.13 versus −0.17, subgroup *P* = 0.05)
Li et al. (2025) [13]	SR & MA	I	TKA	15 RCTs	Single IV dexamethasone 8–16 mg versus placebo	8–16 mg reduced rest- and movement-pain VAS at 24 h (MD −0.71, *P* < 0.001; and −0.89, *P* < 0.001), 24 h morphine consumption (MD −3.06 mg, *P* = 0.001), and length of stay (MD −0.13 days, *P* = 0.03); ROM and recovery improved
Afshar et al. (2025) [14]	Single-center RCT	I	TKA	90	IV dexamethasone 4 mg versus 8 mg versus 16 mg	16 mg was most effective in 48 h pain VAS (4.6 ± 0.7 versus 3.8 ± 0.5 versus 3.6 ± 0.7; *P* < 0.001), 48 h morphine consumption (6.4 ± 1.6 versus 4.2 ± 1.5 versus 4.2 ± 1.4 mg; *P* < 0.001), and sleep quality (*P* = 0.02)
Hannon et al. (2025) [15]	Multicenter RCT	I	TKA	404	IV dexamethasone 4 mg versus 8 mg versus 16 mg	16 mg lowered 24 h opioid use (37.6 versus 37.4 versus 27.0 MME; post hoc 16 versus 8 mg, *P* = 0.047) and rest pain (4.4 versus 5.0 versus 4.1, *P* = 0.02). 16 mg also had fewer 24 h vomiting episodes (0.17 versus 0.18 versus 0.04, *P* = 0.02)
Nielsen et al. (2022) [16]	Multicenter RCT	I	TKA (high pain responders)	84	IV dexamethasone 1.0 mg/kg versus 0.3 mg/kg	Higher dose reduced moderate-to-severe pain (VAS > 30) at 24 h walk (49% versus 79%, *P* < 0.01) and improved recovery
Nielsen et al. (2023) [17]	Multicenter RCT	I	TKA (low pain responders)	157	IV dexamethasone 1.0 mg/kg versus 0.3 mg/kg	No significant difference between doses

*IV* intravenous, *LOE* level of evidence, *MA* meta-analysis, *MD* mean difference, *MME* morphine milligram equivalent, *OR* odds ratio, *POD* postoperative day, *RCT* randomized controlled trial, *ROM* range of motion, *SR* systematic review, *THA* total hip arthroplasty, *TKA* total knee arthroplasty, *VAS* visual analog scale

**Table 3:** Summary of studies on dosing frequency (splitting, repeating, and meta-analytic synthesis)

Study	Study type	LOE	Procedure	*N*	Comparison	Key findings
Lei et al. (2022) [18]	Single-center RCT	I	TKA	192	20 mg versus 10 + 10 mg	Single high dosing gave lower dynamic pain on POD 1–2 (*P* < 0.001, 0.008), while rest pain (*P* = 0.863, 0.991) and morphine (*P* = 0.336) did not differ
Chen et al. (2024) [19]	Single-center RCT	I	TKA	150	10 mg versus 5 + 5 mg	Single and split dosing were comparable in morphine consumption (*P* = 0.543) and rescue analgesia (*P* = 0.538). PONV was lower with split dosing (PONV incidence 20/50 versus 10/50, *P* = 0.029; cumulative PONV score 35 versus 16, *P* = 0.026)
Xu et al. (2018) [20]	Single-center RCT	I	TKA	182	20 mg versus 20 + 10 + 10 mg	Three doses reduced dynamic pain through POD 1–3 versus a single dose (*P* < 0.05), with lower rescue oxycodone (users 10/60 versus 3/61, *P* = 0.037; total 340 versus 50 mg, *P* = 0.028)
Park et al. (2025) [21]	RS	III	TKA	182	10 + 10 mg versus 10 + 10 + 5 mg	Adding a POD 2 dose (3-dose with no IV PCA) maintained pain control comparable to the 2-dose with IV PCA regimen while reducing total opioid use (MME 143.4 versus 108.1, *P* < 0.01), urinary retention (30/89 [34%] versus 13/93 [14%], *P* = 0.003), and POD 1 PONV (0.16 versus 0.01, *P* < 0.01)
Liang et al. (2022) [22]	SR & MA	I	TKA	11 RCTs	Single-dose versus repeat-dose	No significant difference between single and repeat dosing
Hannon et al. (2022) [7]	SR & direct MA	III	TKA + THA	23 studies	Single-dose versus multiple-dose	Multiple doses improved pain control and reduced opioid use
Wang et al. (2025) [23]	SR & MA	I	TKA + THA	12 RCTs	Single-dose versus split-dose versus repeat-dose	Repeat dosing reduced 48 h rest (MD −0.45, *P* < 0.001) and movement (MD −0.69, *P* < 0.001) pain versus a single dose, shortened length of stay (MD −0.28, *P* = 0.004), and lowered rescue analgesia (RR 0.26, *P* = 0.003), with no difference at 24 h. Split dosing was not superior to a single dose (rescue analgesia RR 1.34, *P* = 0.47)

*IV* intravenous, *LOE* level of evidence, *MA* meta-analysis, *MD* mean difference, *MME* morphine milligram equivalent, *PCA* patient-controlled analgesia; *POD* postoperative day, *PONV* postoperative nausea and vomiting, *RCT* randomized controlled trial, *RR* risk ratio, *RS* retrospective study, *SR* systematic review, *THA* total hip arthroplasty, *TKA* total knee arthroplasty

**Table 4:** Summary of studies on safety in the general population

Study	Study type	LOE	Procedure	*N*	Comparison	Key findings
Corcoran et al. (2021) [24] (PADDI trial)	Multicenter RCT	I	Noncardiac surgery	8725	Dexamethasone versus control	No increased SSI risk with 8 mg IV dexamethasone
Asehnoune et al. (2021) [25] (PACMAN trial)	Multicenter RCT	I	Major noncardiac surgery	1222	Dexamethasone versus control	No increase in pneumonia, sepsis, or 14-day mortality
Feeley et al. (2021) [26]	SR & MA	III	TKA + THA	29 studies	Corticosteroid versus control	No increased infection risk across single low-, single high-, or multiple low-dose regimens
Hannon et al. (2022) [7]	SR & Direct MA	III	TKA + THA	23 studies	Corticosteroid versus control	No increase in infection, wound complications, or VTE
Heckmann et al. (2023) [3]	RS (DB)	III	TKA + THA	1,322,025	Dexamethasone versus control	Dexamethasone was associated with a lower risk of PJI in TKA (aOR 0.87, *P* < 0.001) and THA (aOR 0.80, *P* < 0.001)
Vuorinen et al. (2019) [27]	RS (DB)	III	TKA + THA	18,872	Dexamethasone versus control	No significant difference in PJI incidence

*aOR* adjusted odds ratio, *DB* database, *IV* intravenous, *LOE* level of evidence, *MA* meta-analysis, *PJI *periprosthetic joint infection, *RCT* randomized controlled trial, *RS* retrospective study, *SR* systematic review, *SSI* surgical site infection, *THA* total hip arthroplasty, *TKA* total knee arthroplasty, *VTE* venous thromboembolism

**Table 5:** Summary of studies on safety in patients with diabetes (glycemic effects and infection risk)

Study	Study type	LOE	Procedure	*N*	Comparison	Key findings
Park et al (2021) [29]	RS	III	TKA (DM)	427	No dexamethasone versus 5 + 5 mg	Dexamethasone transiently raised blood glucose on the operative day (168.8 versus 204.4 mg/dL, *P* < 0.001) and POD 1 (193.0 versus 210.5 mg/dL, *P* < 0.001) but not beyond POD 1, and a preoperative HbA1c > 7.05% predicted diabetic medication change (AUC 0.811, *P* < 0.001)
Mou et al (2024) [30]	RS	III	TKA + THA (DM)	1,216	No dexamethasone versus 5 mg versus 10 mg	Dexamethasone transiently raised FBG (0/5/10 mg: 10.4/10.7/10.9 mmol/L on POD 0 and 8.7/9.0/9.6 mmol/L on POD 1; all *P* < 0.001) without increasing wound complications or PJI. Preoperative HbA1c (OR 2.843) and FBG (OR 1.412) predicted postoperative FBG > 200 mg/dL (both *P* < 0.001; HbA1c threshold > 7.15%)
Chen et al (2025) [31]	RS	III	TKA (DM)	161	No dexamethasone versus 10 + 10 + 10 mg	Average PBG level and the proportion exceeding 200 mg/dL were comparable between the two groups (10.84 versus 11.05 mmol/L; 43.2% versus 43.9%, *P* = 0.981), with no increase in early complications. Preoperative HbA1c accurately predicted PBG (AUC 0.88, cutoff 6.8%)
Park et al (2025) [32]	Single-center RCT	I	TKA (DM)	83	No dexamethasone versus 10 + 10 mg	Dexamethasone transiently raised blood glucose on the operative day (142.5 versus 159.1 mg/dL, *P* = 0.003) and POD 1 (162.9 versus 186.4 mg/dL, *P* = 0.001), and increased the POD 1 insulin requirement (6.4 versus 9.5 IU, *P* = 0.004)
Tang et al (2025) [33]	Single-center RCT	I	TJA (DM)	135	No dexamethasone versus 10 mg versus 10 + 10 mg	Dexamethasone transiently raised FBG with both single and repeat dosing through POD 2 (mean FBG 141.1/150.4/152.4 mg/dL, *P* = 0.009), normalizing by POD 3 (*P* = 0.44), without increasing the proportion with FBG > 200 mg/dL (15.6% versus 24.4% versus 22.2%, *P* = 0.49) or insulin rescue (*P* = 0.71). Elevated preoperative HbA1c predicted FBG > 200 mg/dL (OR 15.26, *P* < 0.001; threshold 7.3%)
Godshaw et al (2019) [34]	RS	III	TKA + THA	2,317 (657 DM)	No dexamethasone versus 6 or 12 mg in patients with versus without diabetes	Patients with diabetes had a higher PJI rate than patients without diabetes (2.59% versus 0.48%; OR 5.69, *P* < 0.001), but perioperative dexamethasone did not increase PJI overall (*P* = 0.166) or modify infection risk in diabetics (interaction *P* = 0.646)
Jones et al (2024) [35]	RS (DB)	III	TKA + THA	110,568 (34,298 DM)	No dexamethasone versus dexamethasone in patients with versus without diabetes	In patients with diabetes, dexamethasone did not increase the adjusted odds of PJI (TKA aOR 1.1, *P* = 0.36; THA aOR 1.1, *P* = 0.651) or SSI (TKA aOR 0.9, *P* = 0.489; THA aOR 0.7, *P* = 0.197), and significantly lowered the odds of other postoperative infection (UTI, pneumonia, sepsis) (TKA aOR 0.87, *P* = 0.033; THA aOR 0.7, *P* = 0.001)
Kebaish et al (2025) [36]	RS (DB)	III	TKA + THA (DM)	261,474	No dexamethasone versus dexamethasone	Dexamethasone reduced the odds of PJI (aOR 0.82, *P* < 0.001) and sepsis (aOR 0.80, *P* < 0.001) in patients with diabetes, with only a modest increase in postoperative hyperglycemia (aOR 1.14, *P* < 0.001)
Razick et al (2025) [37]	SR & MA	III	TKA + THA (DM)	12 studies	No dexamethasone versus dexamethasone	Dexamethasone raised POD 1 glucose (MD 16.57 mg/dL, *P* = 0.001) with no difference by POD 2–3, and did not increase infection risk (RR 0.82, *P* = 0.61)

*aOR* adjusted odds ratio, *AUC* area under the curve, *DB* database, *DM* diabetes mellitus, *FBG* fasting blood glucose, *HbA1c* glycated hemoglobin, *IU* international unit, *LOE* level of evidence, *MA* meta-analysis, *MD* mean difference, *OR* odds ratio, *PBG* postoperative blood glucose, *PJI* periprosthetic joint infection, *POD* postoperative day, *RCT* randomized controlled trial, *RR* risk ratio, *RS* retrospective study, *SR* systematic review, *SSI* surgical site infection, *THA* total hip arthroplasty, *TJA* total joint arthroplasty, *TKA* total knee arthroplasty, *UTI* urinary tract infection

## Agent and route of administration

Dexamethasone has emerged as the most widely used perioperative corticosteroid in TKA. In the direct meta-analysis by Hannon et al. that informed the 2022 joint clinical practice guidelines, 16 of 23 included studies used dexamethasone as the intervention agent, whereas only 6 used methylprednisolone and 1 used hydrocortisone [4, 7]. This preference is attributable to its pharmacologic profile: dexamethasone has a long biologic half-life of 36–72 h, approximately 25–30 times the anti-inflammatory potency of cortisol, and negligible mineralocorticoid activity, making it well-suited for perioperative use where a sustained anti-inflammatory effect is desired without clinically significant fluid retention [8].

Regarding the route of administration, both IV and periarticular delivery of corticosteroids have been investigated. Hatayama et al. reported in a prospective RCT that periarticular injection of triamcinolone provided superior pain control at 24 h compared with IV dexamethasone [9]. However, in a 4-arm RCT comparing placebo, IV steroid alone, periarticular steroid alone, and their combination, Chan et al. demonstrated that IV administration, either alone or combined with periarticular injection, yielded greater overall pain relief than periarticular injection alone, while the combined regimen provided additional benefits in rehabilitation parameters [10]. A subsequent network meta-analysis of 14 RCTs by Kwak et al. suggested that IV corticosteroids may be preferable for patients at higher risk of PONV, whereas periarticular injection may offer advantages in localized early pain control [11]. Taken together, the current evidence supports IV dexamethasone as the most commonly studied and clinically practical systemic corticosteroid option in TKA. The remainder of this review, therefore, focuses on the clinical questions surrounding systemic dexamethasone use. The summary of the included studies is presented in Table 1.

## Dose optimization

Higher doses of IV dexamethasone have consistently demonstrated superior analgesic outcomes compared with lower doses. Lex et al., in a meta-analysis of 17 RCTs involving TJA, showed that doses exceeding 15 mg were associated with a significantly greater reduction in pain with activity on postoperative day (POD) 0 and 1 [12]. Beyond analgesia, the same meta-analysis found that doses exceeding 15 mg reduced POD 2 PONV relative to placebo. A more recent meta-analysis by Li et al., comparing 15 RCTs and 2584 patients undergoing TKA, confirmed that a single IV dose of 8–16 mg effectively reduced postoperative pain, opioid consumption, and length of hospital stay, while improving range of motion and quality of recovery [13].

Two recent 3-arm RCTs have provided direct dose-comparison data. Afshar et al. randomized patients undergoing TKA to receive 4, 8, or 16 mg of IV dexamethasone postoperatively and found that the 16 mg dose demonstrated the most pronounced effects on pain reduction and sleep quality at 12, 24, and 48 h [14]. Similarly, Hannon et al. compared 4, 8, and 16 mg in a double-blind RCT and reported that 16 mg significantly reduced opioid consumption and pain scores on POD 1 [15]. The 16 mg dose was also associated with fewer vomiting episodes within the first 24 h than 8 or 4 mg (0.04 versus 0.18 versus 0.17, *P* = 0.02), although this antiemetic advantage did not extend to nausea scores or to vomiting beyond 24 h. Based on the available evidence, a fixed IV dose of 16 mg appears to provide greater early analgesic and antiemetic benefit than 4–8 mg in recent TKA RCTs, but the optimal upper dose limit remains undefined.

A fixed-dose approach, however, may not account for individual variability in corticosteroid response. Nielsen et al. conducted two parallel RCTs comparing 1.0 mg/kg with 0.3 mg/kg of IV dexamethasone in patients undergoing TKA stratified by preoperative pain profile. In high pain responders, the higher dose significantly reduced the incidence of moderate to severe pain at 24 h and improved the quality of recovery [16]. By contrast, the same comparison in low pain responders yielded no significant difference [17]. These results highlight the potential value of preoperative risk stratification in tailoring dexamethasone dosing, although further validation in a larger population is warranted. The summary of the included studies is presented in Table 2.

## Dosing frequency

Beyond the question of how much dexamethasone to give, the question of how many times to give is another important point. Two distinct strategies have been investigated: splitting a single total dose across multiple time points and adding supplementary doses to extend the analgesic duration. The summary of the included studies is presented in Table 3.

### Splitting the dose

Several RCTs have examined whether dividing the same total dose improves outcomes compared with single administration. Lei et al. compared a single preoperative dose of 20 mg with a split regimen of 10 mg preoperatively plus 10 mg at 24 h in patients undergoing TKA [18]. The single high-dose regimen produced lower dynamic pain scores than the split regimen on POD 1 and 2, with no difference in resting pain or morphine consumption, leading the authors to conclude that splitting the dose was not superior to a single equivalent dose. Similarly, Chen et al. compared 10 mg as a single dose with 5 mg plus 5 mg and found no difference in opioid consumption, although the split group experienced less PONV [19]. The current evidence, therefore, suggests that splitting the dose offers limited additional analgesic advantage over single-dose administration.

### Repeating the dose

By contrast, adding supplementary doses beyond the initial administration has shown more consistent benefits. Xu et al. demonstrated that extending dexamethasone to three perioperative doses (20 mg preoperatively, 10 mg at 24 h, and 10 mg at 48 h) significantly reduced pain scores through POD 3 compared with a single 20 mg dose [20]. Park et al. further demonstrated that an additional 5 mg dose on POD 2, added to the standard 2-dose regimen of 10 mg on the day of surgery and 10 mg on POD 1, maintained comparable pain levels without IV patient-controlled analgesia while reducing overall opioid consumption and opioid-related side effects, including PONV and urinary retention [21]. Notably, a marked increase in pain scores from POD 1 to 2 was observed in the two-dose group, suggesting that the analgesic effect of dexamethasone wanes by 48 h and that extending coverage beyond this point may be clinically meaningful.

### Meta-analytic synthesis

Meta-analyses have yielded mixed conclusions regarding dosing frequency. Liang et al., in an analysis of 11 RCTs, found no significant subgroup difference between single and repeat dosing regimens [22]. However, the more recent meta-analysis by Hannon et al. of 23 studies concluded that multiple doses improved both pain control and opioid consumption [7]. More recently, Wang et al. conducted a meta-analysis of 12 RCTs specifically comparing single-dose, split-dose, and repeat-dose regimens and found that repeat dosing was superior to both single and split dosing in reducing pain levels, shortening hospitalization, and decreasing the need for rescue analgesia [23]. Collectively, the evidence supports that adding supplementary doses, rather than merely splitting the original dose, provides incremental analgesic benefit, particularly beyond the first 24–48 h. Current evidence favors repeat dosing extended to at least POD 1, with emerging data suggesting that an additional dose on POD 2 may further attenuate the rebound in pain observed after the initial analgesic effect subsides.

## Safety

A major concern surrounding perioperative corticosteroid use has been the potential for immunosuppression-related complications, particularly surgical site infection (SSI) and periprosthetic joint infection (PJI). Reassurance on this issue was first established outside the arthroplasty literature, in large randomized trials of general surgical populations. The PADDI trial, a multicenter RCT enrolling 8,725 patients undergoing noncardiac surgery, found no increased risk of SSI with 8 mg of IV dexamethasone compared with placebo [24]. Similarly, the PACMAN trial, involving 1,222 patients over 50 years of age undergoing major noncardiac surgery, demonstrated that dexamethasone at 0.2 mg/kg did not increase the incidence of pneumonia, sepsis, or 14-day mortality [25]. These trials, however, were conducted in general surgical rather than arthroplasty populations, leaving the question of infection risk specifically after TKA to be addressed by the arthroplasty literature.

Within the arthroplasty literature, the safety profile of perioperative dexamethasone has been extensively evaluated. Feeley et al. conducted a systematic review and meta-analysis of 29 studies and found no increased infection risk with single low-dose, single high-dose, or multiple low-dose corticosteroid regimens in TJA [26]. The direct meta-analysis by Hannon et al. of 23 studies similarly concluded that perioperative corticosteroids were not associated with increased rates of infection, wound complications, or venous thromboembolism [7]. Large database studies have further corroborated these findings. In an analysis of over 1 million contemporary TJA, Heckmann et al. reported that intraoperative dexamethasone was not associated with increased PJI; in fact, the corticosteroid cohort demonstrated a lower infection risk [3]. Vuorinen et al. similarly found no significant difference in PJI incidence among 18,872 arthroplasty patients who received dexamethasone compared with those who did not [27]. Taken together, the current evidence consistently supports the safety of perioperative dexamethasone in TKA with respect to infection and wound complications across a range of doses and regimens. The summary of the included studies is presented in Table 4.

## Safety in patients with diabetes

Despite the reassuring safety data in the general arthroplasty population, the use of perioperative dexamethasone in patients with DM has remained a subject of particular concern, as glucocorticoid-induced hyperglycemia may theoretically increase the risk of postoperative infection. The prevalence of diabetes among patients undergoing TKA is estimated to exceed 20%, making this a clinically important question [28]. The summary of the included studies is presented in Table 5.

### Glycemic effects

Perioperative dexamethasone transiently elevates blood glucose levels in patients with diabetes undergoing TKA. In a retrospective study of 427 patients with diabetes undergoing TKA, Park et al. found that IV dexamethasone caused a temporary increase in postoperative blood glucose and identified a preoperative HbA1c level of 7.05% as a threshold above which patients were more likely to require adjustment of their diabetic medications, regardless of whether dexamethasone was administered [29]. Mou et al. similarly demonstrated a dose-dependent glycemic response, comparing no dexamethasone, 5 mg, and 10 mg in 1,216 patients with diabetic undergoing TJA, and identified preoperative fasting glucose level and HbA1c as independent risk factors for postoperative blood glucose exceeding 200 mg/dL [30]. More recently, Chen et al. confirmed that preoperative HbA1c can reliably predict the magnitude of postoperative blood glucose elevation, providing a practical tool for preoperative risk assessment [31].

To establish prospective evidence in this population, Park et al. conducted an RCT in 83 patients with well-controlled diabetes (HbA1c < 7.0%) undergoing TKA, comparing a repeat-dose regimen of 10 + 10 mg of IV dexamethasone with no dexamethasone [32]. Dexamethasone transiently elevated blood glucose on the day of surgery and POD 1, and increased insulin requirements on POD 1. However, the glycemic changes were resolved promptly, and the dexamethasone group demonstrated significantly reduced postoperative pain. This remains one of the few prospective, randomized trials specifically designed to evaluate both glycemic and analgesic effects of dexamethasone in patients with diabetes undergoing TKA, though its findings apply specifically to well-controlled patients receiving a 10 + 10 mg regimen. Tang et al. subsequently compared single-dose versus repeat-dose dexamethasone in 135 patients with diabetes undergoing TKA and found that both regimens caused a similar transient increase in fasting blood glucose, with no significant difference between single and repeat dosing in the magnitude or duration of glycemic disturbance [33].

### Infection risk in patients with diabetes

While hyperglycemia has been cited as a mechanism by which dexamethasone might increase infection risk in patients with diabetes, large-scale observational data have consistently demonstrated the opposite. Godshaw et al. reviewed 2,317 patients undergoing TJA, of whom 657 had diabetes, and found that although patients with diabetes had a higher baseline infection rate, this was not affected by perioperative dexamethasone administration [34]. Jones et al. conducted a retrospective cohort study of 110,568 patients undergoing TJA, including 34,298 with diabetes, and reported that dexamethasone did not increase the risk of PJI or SSI, and even lowered the risk of other postoperative infection, including pneumonia and sepsis, in patients with diabetes [35]. The average blood glucose increase attributable to dexamethasone was less than 10 mg/dL even in the diabetic cohort, a magnitude unlikely to translate into clinically meaningful infection risk. In the largest study to date, Kebaish et al. analyzed 261,474 patients with diabetes undergoing TJA and found that those who received perioperative dexamethasone had decreased odds of PJI (adjusted odds ratio [aOR] 0.82), further supporting the safety of dexamethasone in this population [36]. A recent systematic review and meta-analysis by Razick et al. synthesized the available evidence and concluded that perioperative dexamethasone appears safe in patients with diabetes [37]. Notably, however, these observational and database studies did not stratify outcomes by dexamethasone dose, and the regimens captured were predominantly in the lower-dose range; they, therefore, do not establish the infection safety of a 16 mg dose in patients with diabetes.

### Dose considerations in patients with diabetes

An important clinical question is whether the dexamethasone dose should be reduced in patients with diabetes to mitigate glycemic excursion. While Mou et al. demonstrated a dose-dependent increase in postoperative blood glucose with escalating dexamethasone doses, large-scale outcome data suggest that this transient hyperglycemia does not translate into increased clinical risk [30]. Jones et al. reported that the average blood glucose increase attributable to dexamethasone was less than 10 mg/dL even in patients with diabetes, and that dexamethasone was associated with lower, not higher, odds of infection [35]. Kebaish et al. confirmed that despite a modest increase in postoperative hyperglycemia (aOR 1.14), patients with diabetes who received dexamethasone had significantly decreased odds of PJI (aOR 0.82) [36]. These findings suggest that the anti-inflammatory benefits of dexamethasone may outweigh the glycemic cost, and that dose reduction based solely on diabetic status is not supported by the current evidence, at least within the 8–10 mg range that constitutes the bulk of the available data. However, it should be noted that most available safety data in patients with diabetes reflect doses of 8–10 mg, or 10 + 10 mg repeat regimens, and are largely confined to well-controlled type 2 diabetes. Whether the higher dose of 16 mg, which has shown superior analgesic efficacy in the general population, or larger cumulative regimens, maintains the same safety profile in patients with diabetes, particularly those with poorly controlled or insulin-dependent diabetes, remains to be established. Until such data are available, a cautious approach favoring 8-10 mg regimens with perioperative glycemic monitoring is reasonable in patients with diabetes.

## Discussion and future directions

Taken together, the current evidence supports IV dexamethasone as an effective component of multimodal analgesia after TKA. In the general TKA population, a 16 mg dose provides greater early analgesic and antiemetic benefit than lower doses. Repeat dosing through POD 1 is well supported, whereas extension to POD 2 remains promising but not yet definitive. With respect to safety, perioperative dexamethasone does not increase the risk of infection or wound complications in the general arthroplasty population. In patients with diabetes, a more conservative strategy favoring 8-10 mg regimens in a well-controlled state with perioperative glycemic monitoring is prudent given the current limits of the evidence. These conclusions should be interpreted in light of the limitations discussed below, and several important knowledge gaps remain.

The optimal dose of dexamethasone in patients with diabetes has not been established. Current dose–response data from the general population support the use of 16 mg for analgesic benefit, but the available safety data in patients with diabetes predominantly reflect doses of 8–10 mg. Whether 16 mg maintains an acceptable glycemic and safety profile in patients with diabetes requires dedicated investigation. Also, the threshold for safe dexamethasone use remains undefined. One of the few prospective RCT in patients with diabetes undergoing TKA enrolled exclusively patients with well-controlled HbA1c < 7.0%, and large database studies demonstrating safety across diabetic populations have not stratified outcomes by HbA1c level [32, 35, 36]. Establishing an evidence-based HbA1c cutoff above which perioperative dexamethasone should be used with heightened caution, or with adjusted monitoring protocols, is a critical priority.

The upper limit of repeat dosing warrants further investigation, particularly from a hypothalamic–pituitary–adrenal (HPA) axis safety perspective. Data from outside the arthroplasty literature suggest that cumulative multiple-dose regimens can lead to partial suppression of adrenocorticotropic hormone (ACTH) and cortisol secretion, with El-Sibai et al. reporting that multiple perioperative doses (cumulative 12–36 mg) produced measurable HPA suppression after the postoperative 15 h, whereas a single dose did not [38]. No study has specifically examined HPA axis dynamics following repeat-dose dexamethasone protocols in TKA, and the threshold at which cumulative dosing may produce clinically meaningful adrenal suppression is unknown.

The role of oral dexamethasone as an alternative to IV administration is also emerging. With the growing trend toward ambulatory TKA, oral formulations offer a practical advantage after hospital discharge. Recent RCTs have demonstrated the efficacy of oral dexamethasone in a dose-dependent manner, and its oral bioavailability of approximately 70–80% makes dose conversion pharmacologically feasible [39, 40]. However, no head-to-head trial has directly compared oral and IV dexamethasone in TKA, and this comparison is needed to guide outpatient protocols.

## Limitations

This review has several limitations. First, it was conducted as a clinically oriented narrative synthesis rather than a formal systematic review. Although a structured, comprehensive literature search of multiple databases with predefined inclusion and exclusion criteria was performed, this review did not undertake preferred reporting items for systematic reviews and meta-analyses (PRISMA)-guided screening, duplicate study selection, or a comprehensive risk-of-bias assessment of all included studies; methodological quality was instead indicated by the level of evidence reported for each study in Tables 1, 2, 3, 4, and 5, and appraised for the pivotal primary studies underlying principal recommendations. The synthesis was also restricted to English-language publications, which may introduce language bias. Second, the underlying evidence base is heterogeneous. The included studies differed considerably in their multimodal analgesic protocols and in the dose, route, and timing of dexamethasone administration, which limits direct comparison across trials and precludes quantitative pooling. Several studies reported combined total joint arthroplasty cohorts rather than isolated TKA, so knee-specific effects could not always be distinguished from those in total hip arthroplasty. Third, evidence for the principal dosing and safety questions remains incomplete. Individual RCTs were generally underpowered to detect differences in rare infectious end points such as PJI and SSI, and the reassuring infection data, therefore, derive largely from observational and database studies that remain subject to residual confounding despite statistical adjustment. The optimal upper dose limit likewise remains unclear. Finally, evidence in patients with diabetes is the most constrained. Available safety data predominantly reflect single or repeated doses of 8–10 mg in patients with well-controlled type 2 diabetes, and the safety of the higher 16 mg dose, of larger cumulative regimens, and of dexamethasone use in poorly controlled or insulin-dependent diabetes remains insufficiently established. A validated HbA1c threshold above which perioperative dexamethasone should be avoided or more closely monitored has not yet been defined.

## Conclusions

Current evidence supports IV dexamethasone as an effective component of multimodal analgesia after TKA. In the general TKA population, 16 mg appears more effective than lower doses for early postoperative pain, opioid consumption, and PONV-related outcomes. Repeat dosing through POD 1 is supported by available evidence, whereas extension to POD 2 remains promising but requires further validation. In patients with well-controlled diabetes, perioperative dexamethasone appears safe with appropriate glycemic monitoring, although safety data are largely limited to doses of 8–10 mg, and the safety of the 16 mg dose in this population remains insufficiently defined.

## Supplementary information

Supplementary material 1.

Supplementary material 2.

Supplementary material 3.

## Data Availability

No datasets were generated or analyzed during the current study.
