# Supplementary material for: Optimizing perioperative systemic dexamethasone use in total knee arthroplasty: a narrative review on current evidence
Source: Knee Surg Relat Res. 2026 Aug 3;38:36. doi: 10.1186/s43019-026-00336-2 (PMC13430760; doi:10.1186/s43019-026-00336-2)
Supplement: Supplementary file 1 — Supplementary material 1. [file 43019_2026_336_MOESM1_ESM.docx]

**Supplementary File 1. Detailed Search Strategy**

The following search strategies were used across three databases. All searches were conducted in April 2026 and limited to articles published between January 2015 and April 2026.

***Database 1: PubMed/MEDLINE***
The search combined three conceptual blocks using Boolean operators:

Block 1 (Population): "total knee arthroplasty" OR "total knee replacement" OR "TKA" OR "total knee"

Block 2 (Intervention): "dexamethasone" OR "corticosteroid" OR "glucocorticoid" OR "methylprednisolone" OR "triamcinolone" OR "betamethasone" OR "hydrocortisone" OR "steroid"

Block 3 (Outcomes): "pain" OR "analgesia" OR "analgesic" OR "dose" OR "dosing" OR "infection" OR "surgical site infection" OR "periprosthetic joint infection" OR "diabetes" OR "diabetic" OR "hyperglycemia" OR "blood glucose" OR "glycemic" OR "HbA1c" OR "safety" OR "complication" OR "nausea" OR "vomiting" OR "PONV" OR "opioid" OR "multimodal"

Final search: Block 1 AND Block 2 AND Block 3

Filters: English language; Humans; Publication date 2015/01/01 to 2026/04/30

***Database 2: EMBASE***
The same conceptual blocks were adapted for EMBASE syntax using Emtree terms and free-text keywords:

Block 1: 'total knee arthroplasty'/exp OR 'total knee replacement' OR 'TKA'

Block 2: 'dexamethasone'/exp OR 'corticosteroid'/exp OR 'glucocorticoid'/exp OR 'methylprednisolone'/exp OR 'triamcinolone'/exp

Block 3: 'pain'/exp OR 'analgesia'/exp OR 'dose' OR 'infection'/exp OR 'diabetes mellitus'/exp OR 'hyperglycemia'/exp OR 'safety'/exp

Final search: Block 1 AND Block 2 AND Block 3

Filters: English language; Humans; 2015–2026; Exclude conference abstracts

***Database 3: Cochrane Central Register of Controlled Trials (CENTRAL)***
The search used free-text terms:

Block 1: "total knee arthroplasty" OR "total knee replacement" OR "TKA"

Block 2: "dexamethasone" OR "corticosteroid" OR "glucocorticoid" OR "steroid"

Block 3: "pain" OR "dose" OR "infection" OR "diabetes" OR "safety"

Final search: Block 1 AND Block 2 AND Block 3

Filters: 2015–2026; Trials only
