# Supplementary figures and images for: Optimizing perioperative systemic dexamethasone use in total knee arthroplasty: a narrative review on current evidence
Source: Knee Surg Relat Res. 2026 Aug 3;38:36. doi: 10.1186/s43019-026-00336-2 (PMC13430760; doi:10.1186/s43019-026-00336-2)

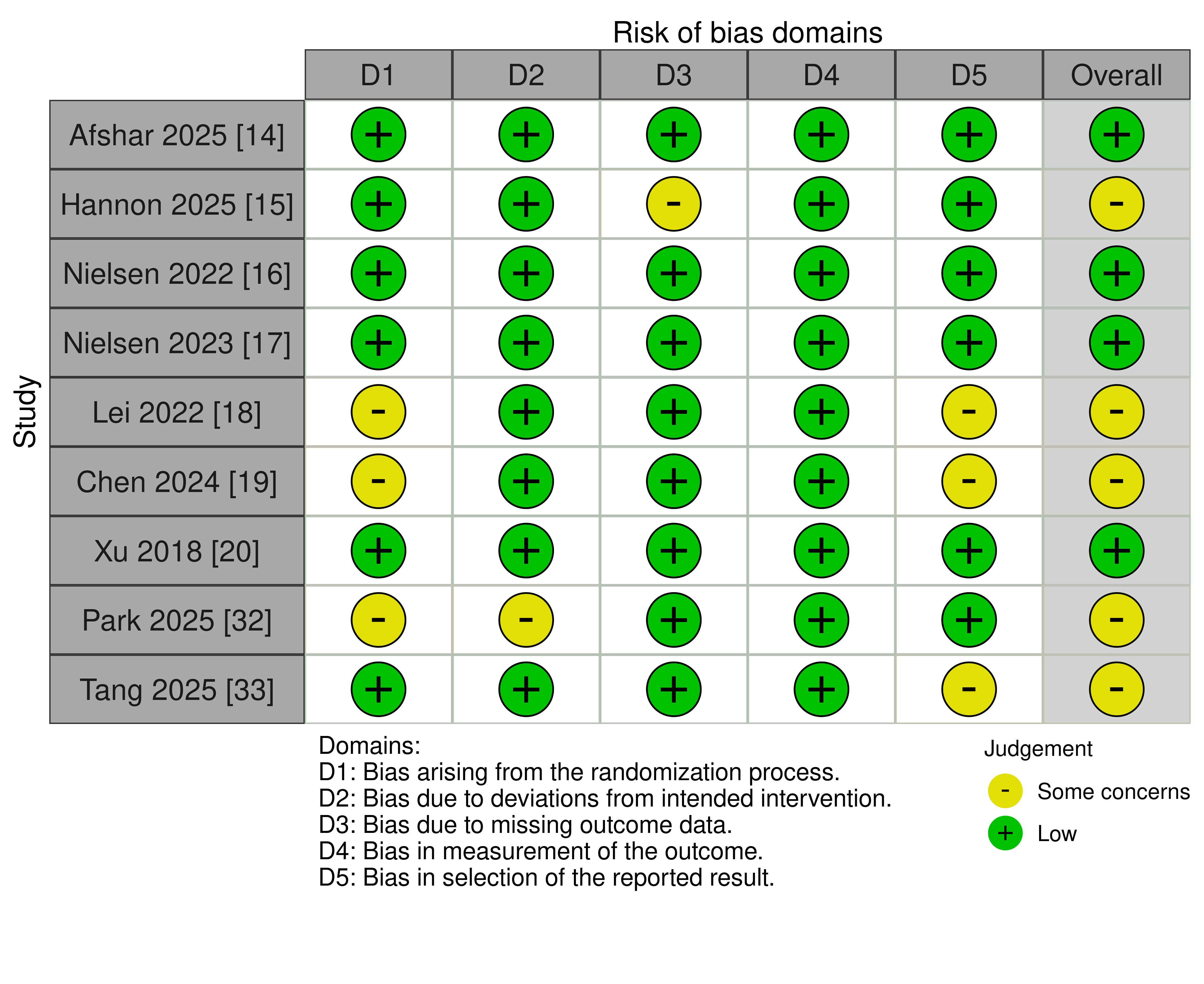

Supplement: Supplementary file 2 — Supplementary material 2. [file 43019_2026_336_MOESM2_ESM.png]

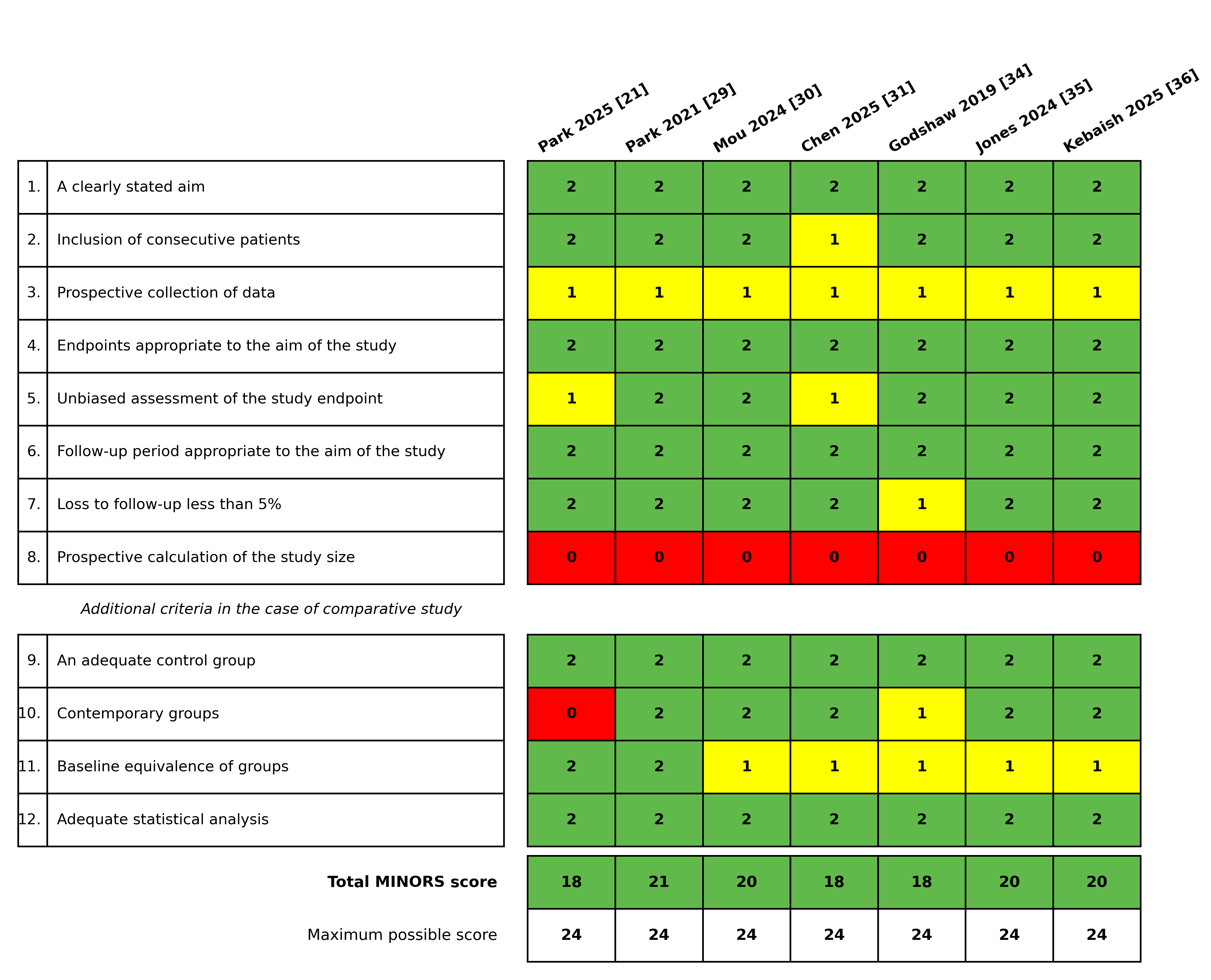

Supplement: Supplementary file 3 — Supplementary material 3. [file 43019_2026_336_MOESM3_ESM.png]
